# Association of Admission Hyponatremia With Clinical Severity and In-Hospital Outcomes in ST-Elevation Myocardial Infarction: A Single-Centre Retrospective Observational Study

**DOI:** 10.7759/cureus.109970

**Published:** 2026-05-31

**Authors:** Alpana Meena, Yashoverdhan Kag, Vikas Sahu, Gunjalika Kag

**Affiliations:** 1 General Medicine, Lakshmi Narain (LN) Medical College and Research Center, Bhopal, IND

**Keywords:** disease mortality, electrolyte imbalance, #hyponatremia, killip class, left ventricular ejection fraction, retrospective research, st-elevation myocardial infarction (stemi)

## Abstract

Introduction

Hyponatremia, defined as a serum sodium concentration below 135 mEq/L, is the most common electrolyte disorder encountered in clinical practice. In the context of ST-elevation myocardial infarction (STEMI), haemodynamic stress, neurohormonal activation, and diuretic use may predispose patients to hyponatremia. The precise relationship between hyponatremia and clinical severity in STEMI remains incompletely characterised, particularly in South Asian populations. The objective of the study is to determine the prevalence of hyponatremia in patients admitted with STEMI and to evaluate its association with markers of disease severity and in-hospital mortality.

Methods

A single-centre retrospective observational study reviewed 196 consecutive STEMI admissions. Hyponatremia was defined as serum sodium <135 mEq/L on admission. Clinical severity was assessed using the Killip classification, left ventricular ejection fraction (LVEF), cardiogenic shock, and ICU length of stay. In-hospital mortality was the primary outcome. Comparisons used the independent samples t-test, chi-square test, and one-way ANOVA, with Spearman rank correlation and parsimonious logistic regression as secondary analyses.

Results

Hyponatremia was identified in 54 of 196 patients (27.6%). Binary hyponatremia classification was not significantly associated with categorical severity endpoints or mortality; however, lower serum sodium levels demonstrated a weak but significant inverse correlation with ICU stay duration (Spearman r = −0.156; p = 0.030). The left circumflex artery (LCX) was significantly more frequent as the culprit vessel in hyponatraemic patients (11/54, 20.4% vs 6/142, 4.2%; p = 0.002). Serum sodium levels demonstrated statistically significant variation across Killip categories without a clear monotonic severity gradient (one-way ANOVA: F = 5.830; p = 0.001). Hyponatremia was not an independent predictor of in-hospital mortality on logistic regression (OR 1.08; 95% CI 0.39-3.02; p = 0.884).

Conclusion

Hyponatremia affects 54 of 196 patients (27.6%) in this STEMI cohort. Binary hyponatremia classification was not significantly associated with categorical severity endpoints or in-hospital mortality; however, lower serum sodium levels demonstrated a weak but significant inverse correlation with ICU stay duration, and LCX-territory infarcts were more prevalent among hyponatraemic patients. Prospective multicentre studies are warranted.

## Introduction

ST-elevation myocardial infarction (STEMI) remains a leading cause of morbidity and mortality worldwide, with a substantial burden in low- and middle-income countries including India [1]. Despite advances in reperfusion therapy, risk stratification relies predominantly on clinical and echocardiographic parameters such as the Killip classification and left ventricular ejection fraction.

Hyponatremia, defined as a serum sodium concentration below 135 mEq/L, is the most prevalent electrolyte abnormality in hospitalised patients, with a reported prevalence of 15-30% in acute coronary syndrome (ACS) populations [2,3]. In STEMI, its pathophysiology is multifactorial, involving non-osmotic antidiuretic hormone (ADH) release driven by reduced cardiac output, renin-angiotensin-aldosterone system (RAAS) activation, and the haemodynamic effects of intravenous fluids and diuretics administered during resuscitation [4]. The relationship between hyponatremia and adverse clinical outcomes has been well documented in heart failure [5,6], and emerging evidence suggests a similar prognostic significance in the setting of ACS [7,8].

Retrospective studies in Western populations have associated admission hyponatremia with higher Killip class, greater haemodynamic compromise, and increased short- and long-term mortality in acute myocardial infarction [3,8,9]. However, data from South Asian cohorts remain limited, and the association between hyponatremia and STEMI severity in patients managed in an Indian tertiary care setting has not been systematically examined. In particular, few studies have explored angiographic correlations or evaluated admission sodium as a continuous variable in relation to severity markers and ICU stay in Indian STEMI cohorts.

The primary objective of this retrospective observational study was to evaluate the association between admission hyponatremia and in-hospital mortality in a South Asian STEMI cohort managed with primary PCI. Pre-specified secondary objectives included assessment of hyponatremia prevalence and its association with established clinical severity markers - Killip classification, left ventricular ejection fraction (LVEF), cardiogenic shock, and ICU stay duration - and evaluation of serum sodium as a continuous variable across haemodynamic severity strata. The relationship between hyponatremia and culprit vessel distribution was examined as an exploratory post-hoc analysis.

## Materials and methods

Study design and setting

This was a single-centre, retrospective observational study conducted at LN Medical College & Research Centre, Bhopal, Madhya Pradesh, India. Medical records of patients admitted with a confirmed diagnosis of STEMI were reviewed for the period October 2024- October 2025. Ethical approval was obtained from the Institutional Ethics Committee (Reference: LNMC&RC/Dean/2025/Ethics/273), and the study was conducted in accordance with the Declaration of Helsinki. Given the retrospective nature of the study, the requirement for individual informed consent was waived by the ethics committee.

Study population

Medical records of 196 consecutive patients with a diagnosis of STEMI were reviewed. STEMI was defined according to the 2017 ESC guidelines as persistent ST-segment elevation ≥1 mm in at least two contiguous limb leads or ≥2 mm in precordial leads, or new left bundle branch block, in the context of symptoms consistent with acute myocardial ischaemia [10]. Patients with pre-existing chronic hyponatremia of known aetiology (hypothyroidism, cirrhosis, nephrotic syndrome, or syndrome of inappropriate ADH secretion), age below 18 years, and those with incomplete clinical records were excluded.

Data extraction

Demographic data (age, sex), comorbidities (hypertension, diabetes mellitus, thyroid disease), and clinical parameters were extracted from hospital records. Laboratory investigations, including serum electrolytes (sodium, potassium), renal function tests (urea, creatinine), and cardiac troponin, were recorded from admission blood samples obtained at initial presentation prior to major in-hospital therapeutic interventions, wherever feasible within routine clinical workflow. Transthoracic echocardiography reports were reviewed for LVEF assessment. Culprit-vessel assignment was based on coronary angiography findings documented in procedural records. Killip class at admission, ICU length of stay, and in-hospital outcome (discharge or death) were documented.

Definitions

Hyponatremia was defined as a serum sodium concentration <135 mEq/L measured on the admission blood sample [11]. The Killip classification, originally described by Killip and Kimball in 1967 [12], is a bedside clinical tool used to stratify the severity of heart failure in patients with acute myocardial infarction based on physical examination findings. Class I denotes no clinical signs of heart failure (absence of pulmonary rales, third heart sound, or elevated jugular venous pressure); Class II indicates mild-to-moderate heart failure with basal pulmonary rales, third heart sound (S3), or elevated jugular venous pressure; Class III represents acute pulmonary oedema with rales extending beyond the lung bases; and Class IV denotes cardiogenic shock characterised by systolic blood pressure below 90 mmHg with signs of peripheral hypoperfusion including oliguria, cyanosis, and diaphoresis. Cardiogenic shock was defined as sustained hypotension (systolic blood pressure <90 mmHg for ≥30 minutes) with evidence of end-organ hypoperfusion. Prolonged ICU stay was defined as an intensive care unit admission exceeding seven days.

Statistical analysis

Continuous variables are expressed as mean ± standard deviation (SD); categorical variables are expressed as frequencies and percentages. Normality of distribution was assessed using the Shapiro-Wilk test. For normally distributed continuous variables, the independent samples t-test was used for between-group comparisons, and results are reported as t-values with corresponding p-values. Categorical variables were compared using the chi-square test (reported as χ² values) or Fisher's exact test where appropriate. One-way ANOVA (reported as F-values) was used to compare serum sodium levels across Killip classes; findings were corroborated by non-parametric analysis. Spearman's rank correlation coefficients were calculated to assess the relationship between serum sodium and clinical severity parameters. A parsimonious logistic regression model - comprising hyponatremia status, age, diabetes mellitus, and serum creatinine - was used to evaluate independent predictors of in-hospital mortality. This variable set was selected a priori, given the limited number of in-hospital mortality events (n = 20), to avoid overfitting. Given the limited mortality event count, the regression model was intentionally restricted to a small number of clinically relevant covariates to minimise overfitting. Results are reported as odds ratios (OR) with 95% confidence intervals (CI) and Wald z-statistics. All statistical analyses were performed using Python (version 3.10) (Python Software Foundation, Fredericksburg, VA, USA) with the SciPy and scikit-learn libraries. A two-tailed p-value <0.05 was considered statistically significant.

## Results

Study population and prevalence of hyponatremia

A total of 196 STEMI patients were included in the retrospective analysis. The mean age of the cohort was 61.0 ± 11.7 years, and 150 patients (76.5%) were male. The mean serum sodium at admission was 138.4 ± 6.6 mEq/L (range: 121-158 mEq/L). Hyponatremia was identified in 54 of 196 patients (27.6%). Anterior STEMI was the predominant MI type (93/196, 47.4%), followed by inferior STEMI (84/196, 42.9%).

Baseline characteristics

Baseline demographic and laboratory characteristics stratified by sodium status are presented in Table 1.

**Table 1:** Baseline Characteristics Stratified by Sodium Status Na = sodium; SD = standard deviation; RCA = right coronary artery; LAD = left anterior descending artery; LCX = left circumflex artery. t-test used for continuous variables (reported as t-value); chi-square test for categorical variables (reported as χ²). *p < 0.05 (statistically significant). The χ² and p-value for culprit vessel applies to the overall three-category distribution (RCA/LAD/LCX).

Variable	Hyponatremia (n=54)	Normal Na (n=142)	Test statistic	p-value
Age (years), mean ± SD	59.7 ± 10.0	61.4 ± 12.2	t = −0.920	0.359
Male sex, n (%)	41 (75.9%)	109 (76.8%)	χ² = 0.000	1.000
Hypertension, n (%)	29 (53.7%)	89 (62.7%)	χ² = 0.967	0.326
Diabetes mellitus, n (%)	24 (44.4%)	56 (39.4%)	χ² = 0.225	0.635
Thyroid disease, n (%)	3 (5.6%)	15 (10.6%)	χ² = 0.653	0.419
Serum potassium (mEq/L), mean ± SD	4.54 ± 0.55	4.41 ± 0.58	t = 1.387	0.167
Serum urea (mg/dL), mean ± SD	52.2 ± 21.5	48.8 ± 19.3	t = 1.074	0.284
Serum creatinine (mg/dL), mean ± SD	1.26 ± 0.50	1.30 ± 0.52	t = −0.505	0.614
Anterior MI, n (%)	24 (44.4%)	69 (48.6%)	χ² = 0.129	0.719
Inferior MI, n (%)	25 (46.3%)	59 (41.5%)	χ² = 0.192	0.661
Positive troponin, n (%)	48 (88.9%)	108 (76.1%)	χ² = 3.216	0.073
Culprit vessel distribution, n (%)	54 (100%)	142 (100%)	χ² = 12.899	0.002*
RCA	18 (33.3%)	55 (38.7%)	—	—
LAD	25 (46.3%)	81 (57.0%)	—	—
LCX	11 (20.4%)	6 (4.2%)	—	—

No significant differences were observed between groups for age, sex, comorbidities, or renal function parameters (all p > 0.05). A significant finding was the higher prevalence of the left circumflex artery (LCX) as culprit vessel in the hyponatremia group compared to the normonatremia group (11/54, 20.4% vs 6/142, 4.2%; p = 0.002).

Clinical severity and outcomes

Table 2 presents clinical severity parameters and in-hospital outcomes.

**Table 2:** Clinical Severity and In-Hospital Outcomes by Sodium Status LVEF = left ventricular ejection fraction. t-test used for continuous variables (reported as t-value); chi-square test for categorical variables (reported as χ²). The χ² for Killip class rows reflects the overall four-category distribution and is reported uniformly across class rows.

Variable	Hyponatremia (n=54)	Normal Na (n=142)	Test statistic	p-value
Ejection fraction (%), mean ± SD	41.1 ± 14.7	38.9 ± 11.8	t = 1.114	0.267
Killip class I, n (%)	24 (44.4%)	74 (52.1%)	χ² = 7.367	0.061
Killip class II, n (%)	17 (31.5%)	21 (14.8%)	χ² = 7.367	0.061
Killip class III, n (%)	5 (9.3%)	22 (15.5%)	χ² = 7.367	0.061
Killip class IV, n (%)	8 (14.8%)	25 (17.6%)	χ² = 7.367	0.061
Severe Killip class (III + IV), n (%)	13 (24.1%)	47 (33.1%)	χ² = 1.105	0.293
Cardiogenic shock, n (%)	8 (14.8%)	23 (16.2%)	χ² = 0.000	0.986
ICU stay > 7 days, n (%)	38 (70.4%)	85 (59.9%)	χ² = 1.427	0.232
In-hospital mortality, n (%)	6 (11.1%)	14 (9.9%)	χ² = 0.000	1.000

Mean LVEF was comparable between groups (41.1 ± 14.7% vs 38.9 ± 11.8%; p = 0.267). Killip class distribution showed a numerically greater proportion of class II in the hyponatremia group (17/54, 31.5% vs 14.8%), but the overall distribution did not reach statistical significance (p = 0.061). Severe Killip class (III+IV) was present in 13 of 54 hyponatraemic patients (24.1%) versus 47 of 142 normonatremia patients (33.1%; p = 0.293). Binary hyponatremia classification was not significantly associated with categorical severity endpoints or in-hospital mortality; however, lower serum sodium levels demonstrated a weak but significant inverse correlation with ICU stay duration (Spearman r = −0.156; p = 0.030), detailed further in the correlation analysis below. Twenty in-hospital deaths occurred in total: 6 of 54 patients (11.1%) in the hyponatremia group and 14 of 142 patients (9.9%) in the normonatremia group (p = 1.000).

Serum sodium across Killip classes

When serum sodium was analysed as a continuous variable across Killip classes (Table 3), serum sodium levels demonstrated statistically significant variation across Killip categories without a clear monotonic severity gradient (one-way ANOVA: F = 5.830; p = 0.001; corroborated by non-parametric analysis).

**Table 3:** Serum Sodium Levels and Hyponatremia Prevalence by Killip Class Na = sodium; SD = standard deviation; HF = heart failure. One-way ANOVA F-value (5.830) and p-value (0.001) for the comparison of mean serum sodium across all four Killip classes are reported once in the first data row; — = not repeated for subsequent rows. *p < 0.05.

Killip Class	n	Mean Na (mEq/L)	SD	Hyponatremia n (%)	F-value	p-value
Class I — No heart failure	98	138.5	6.1	24 (24.5%)	5.830	0.001*
Class II — Mild heart failure	38	135.1	6.9	17 (44.7%)	—	—
Class III — Pulmonary oedema	27	141.4	6.5	5 (18.5%)	—	—
Class IV — Cardiogenic shock	33	139.6	6.6	8 (24.2%)	—	—

The lowest mean sodium was observed in Killip class II patients (135.1 ± 6.9 mEq/L; 17/38 patients, 44.7% with hyponatremia), rather than in the most severely ill class IV patients (139.6 ± 6.6 mEq/L; 8/33 patients, 24.2% with hyponatremia).

Correlation and logistic regression analyses

Spearman's rank correlation demonstrated no significant association between serum sodium and Killip class (r = 0.058; p = 0.422), LVEF (r = −0.032; p = 0.654), or in-hospital mortality (r = −0.063; p = 0.381). A weak but statistically significant inverse correlation was observed between serum sodium and ICU stay duration (Spearman r = −0.156; p = 0.030), indicating that lower admission sodium levels were associated with a more prolonged intensive care course.

Table 4 presents the results of the parsimonious logistic regression model.

**Table 4:** Logistic Regression Analysis — Independent Predictors of In-Hospital Mortality OR = odds ratio; CI = confidence interval. Model includes hyponatremia status, age, diabetes mellitus, and serum creatinine. Variable selection was a priori given the limited event count (n = 20 deaths). Wald z-statistic reported.

Variable	OR	95% CI	Wald z	p-value
Hyponatremia (Na < 135 mEq/L)	1.08	0.39 – 3.02	0.15	0.884
Age (per year)	0.97	0.93 – 1.01	−1.50	0.133
Diabetes mellitus	1.59	0.62 – 4.10	0.96	0.337
Creatinine (per mg/dL)	0.40	0.15 – 1.04	−1.88	0.059

Hyponatremia was not identified as an independent predictor of in-hospital mortality (OR 1.08; 95% CI 0.39-3.02; p = 0.884). No variable in the model reached statistical significance, with serum creatinine showing the strongest - though non-significant - inverse association with mortality (OR 0.40; 95% CI 0.15-1.04; p = 0.059).

## Discussion

This single-centre retrospective observational study examined the association between admission hyponatremia and clinical severity and in-hospital mortality in 196 consecutive STEMI patients. The principal findings are: (i) hyponatremia was present in 54 of 196 patients (27.6%); (ii) binary hyponatremia classification was not significantly associated with categorical severity endpoints or mortality; (iii) lower serum sodium levels demonstrated a weak but statistically significant inverse correlation with ICU stay duration (r = −0.156; p = 0.030); (iv) serum sodium levels demonstrated statistically significant variation across Killip categories without a clear monotonic severity gradient (F = 5.830; p = 0.001), with the lowest mean sodium in Killip class II; and (v) 11 of 54 hyponatremic patients (20.4%) had LCX as the culprit vessel compared to 6 of 142 hyponatraemic patients (4.2%; p = 0.002).

The 27.6% prevalence of hyponatremia is consistent with reported rates of 15-30% in ACS populations [2,3,7]. The pathophysiology involves neurohormonal activation - particularly non-osmotic ADH secretion and RAAS stimulation - in response to reduced effective arterial blood volume [4]. The well-established adverse prognostic role of hyponatremia in heart failure [5,6] has led to increasing interest in its significance across other cardiovascular disease states; a systematic review and meta-analysis by Ma et al. reported that hyponatremia was significantly associated with increased 30-day mortality (RR 2.18; 95% CI 1.96-2.42) in ACS patients [7]. The observation that 11 of 54 hyponatraemic patients (20.4%) had LCX as the culprit vessel compared to 6 of 142 normonatremic patients (4.2%) is a novel finding. The observed LCX association should be considered exploratory and hypothesis-generating. Although regional infarct characteristics may potentially influence neurohormonal activation patterns, the present dataset was not designed to establish mechanistic relationships, and this finding requires prospective validation.

The weak inverse correlation between serum sodium and ICU stay duration (r = −0.156; p = 0.030) suggests a possible relationship between lower sodium levels and prolonged intensive care requirement, although the modest effect size and retrospective design limit definitive prognostic interpretation. Plakht et al. similarly reported that sodium levels during hospitalisation for acute myocardial infarction were markers of in-hospital adverse outcomes [13], and Tada et al. demonstrated that early hyponatremia in STEMI was associated with both short- and long-term adverse outcomes [14].

The ANOVA finding of significant sodium variation across Killip classes (F = 5.830; p = 0.001) requires careful contextualisation. Serum sodium levels demonstrated statistically significant variation across Killip categories without a clear monotonic severity gradient - the lowest mean sodium was observed in Killip class II rather than class IV. The observed non-linear variation in sodium across Killip classes should also be interpreted cautiously. While differential neurohormonal activation across severity stages may contribute, the absence of a monotonic gradient and the limited sample size preclude definitive mechanistic inference.

Hyponatremia was not identified as an independent predictor of in-hospital mortality in the present cohort (OR 1.08; 95% CI 0.39-3.02; p = 0.884); however, this finding should be interpreted cautiously given the limited number of mortality events (n = 20), which substantially reduced statistical power and may have limited the ability to detect modest prognostic associations. The non-significant inverse trend for creatinine (OR 0.40; p = 0.059) is consistent with the established prognostic role of renal impairment in STEMI and warrants evaluation in a larger cohort. The discordance with some larger Western studies associating hyponatremia with STEMI mortality [3,8,9] may reflect differences in sample size, patient demographics, reperfusion protocols, or concurrent medication use.

Limitations

Several limitations merit acknowledgement. First, the moderate sample size (n = 196) with only 20 in-hospital deaths limits statistical power, particularly for regression modelling. Second, the retrospective design precluded standardised collection of pre-admission and early in-hospital medication data - particularly diuretics, ACE inhibitors, ARBs and intravenous fluid administration - that may significantly influence admission sodium levels. Baseline volume status and pre-existing subclinical heart failure severity could not be comprehensively assessed. Third, sodium was measured at a single time point; serial measurements would better capture the dynamic neurohormonal response to STEMI. Fourth, residual confounding related to reperfusion strategy, medication exposure, and fluid administration cannot be excluded. Fifth, exclusion of patients with chronic hyponatremia of known aetiology may introduce selection bias. Lastly, the single-centre design limits generalisability, and residual confounding from unmeasured variables cannot be excluded.

## Conclusions

Hyponatremia was common among STEMI patients in this retrospective cohort. Binary hyponatremia classification was not significantly associated with categorical severity endpoints or in-hospital mortality. However, lower serum sodium levels demonstrated a weak inverse association with ICU stay duration, while exploratory associations with culprit-vessel distribution and Killip-category sodium variation were observed. These findings suggest that admission sodium may represent a limited exploratory marker of clinical severity, warranting further prospective multicentre investigation with serial sodium assessment and improved adjustment for treatment-related confounders.
